# A Novel Method for Remote Depth Estimation of Buried Radioactive Contamination

**DOI:** 10.3390/s18020507

**Published:** 2018-02-08

**Authors:** Ikechukwu Kevin Ukaegbu, Kelum A. A. Gamage

**Affiliations:** 1 Engineering Department, Lancaster University, Lancaster LA1 4YW, UK; 2 School of Engineering, University of Glasgow, Glasgow G12 8QQ, UK; Kelum.Gamage@glasgow.ac.uk

**Keywords:** remote depth profiling, radiation detection, radioactive contamination, radiological characterisation, nuclear wastes, nuclear decommissioning

## Abstract

Existing remote radioactive contamination depth estimation methods for buried radioactive wastes are either limited to less than 2 cm or are based on empirical models that require foreknowledge of the maximum penetrable depth of the contamination. These severely limits their usefulness in some real life subsurface contamination scenarios. Therefore, this work presents a novel remote depth estimation method that is based on an approximate three-dimensional linear attenuation model that exploits the benefits of using multiple measurements obtained from the surface of the material in which the contamination is buried using a radiation detector. Simulation results showed that the proposed method is able to detect the depth of caesium-137 and cobalt-60 contamination buried up to 40 cm in both sand and concrete. Furthermore, results from experiments show that the method is able to detect the depth of caesium-137 contamination buried up to 12 cm in sand. The lower maximum depth recorded in the experiment is due to limitations in the detector and the low activity of the caesium-137 source used. Nevertheless, both results demonstrate the superior capability of the proposed method compared to existing methods.

## 1. Introduction

A significant amount of radioactive waste is generated during the life cycle of a typical nuclear facility e.g., nuclear power plant [1]. These wastes can be by-products of radioactive materials such as nuclear fuels or previously non-radioactive materials that become contaminated either through contact with radioactive materials or through activation by ionising radiation. Characterisation of these wastes is critical in decommissioning these facilities because it provides vital information required for effective planning, dismantling, transporting and storage of these wastes to meet nuclear regulatory standards [2,3].

A key step in the characterisation process is the localisation of these wastes [4]. However, some of these wastes can be in difficult to access areas, which causes their localisation to be particularly challenging. Examples of such wastes commonly encountered during decommissioning of nuclear facilities include wastes buried inside porous materials such as concrete and soil. The contamination of concrete structures is usually due to ingress of radioactive contaminants as result of irradiation or leaks and spills [1]. Furthermore, these contaminants can also interact with the constituent of the concrete resulting in cracks that allow the contaminants to penetrate deeper into the concrete structure over time [5]. There are several pathways through which anthropogenic radiological contaminants can end up in the soil. This include leaks from underground waste transportation pipes and storage pounds [6], deliberate burial of wastes in the soil [7] and particles from radiological fallouts that precipitate into the soil [8]. For instance, the reported contamination at the beaches of Northern Scotland covers an area of about 200,000 m${}^{2}$ and consists mainly of caesium-137 fuel fragments with activities of up to $10^{8}$ Bq buried at depths of less than 1 m [9,10].

The major difficulty in localising wastes buried in concrete or soil is the determination of the depth of penetration of the contamination. This is because of the visually opaque nature of these porous materials. However, knowledge of the depth of penetration of these contaminants is vital in choosing the most cost-effective decommissioning strategy. For instance, decommissioning concrete structures is usually a trade-off between scarification and designation of the entire concrete structure as waste [11]. However, scarification is expensive and time wasting if the contamination is found to have penetrated deeper than expected. On the other hand, designation of the entire concrete structure as wastes significantly increases the volume and cost of wastes to be disposed if the contamination is shallow. Therefore, the importance of effective depth profiling methods for entrained contamination cannot be over emphasised. Traditional depth profiling methods include: Logging, Micro drilling and Core sampling [7,12]. However, these methods are destructive and time-consuming. In addition, they also have limited spatial extent for sampling.

Consequently, various remote depth profiling methods have been investigated and reported in literature. These include: the relative attenuation method [13,14,15] and principal component analysis (PCA) method [16,17,18]. The relative attenuation method exploits the relative difference in the attenuation of two prominent peaks (typically the X-ray and gamma photo peaks) in the measured energy spectrum of the buried radionuclide. However, the use of the X-ray photo peak limits the maximum detectable depth to less than 2 cm due to high attenuation of the X-rays. Furthermore, the technique is not effective for radionuclides such as cobalt-60 (Co-60) that do not emit sufficient X-rays [14]. The PCA method is based on a nonlinear regression model that correlates a derived variable referred to as the synthetic angle with the depth of the buried radionuclide. The synthetic angle was defined as the inverse tangent of the ratio of the first two principal components of a set of measured spectra of the radioactive source for different burial depths. However, such empirical models are data dependent. Consequently, the model parameters change whenever a new spectra is added to the original data [18]. This makes the model useful only when the maximum penetrable depth of the contamination is known a priori.

Therefore, this paper presents a novel remote depth estimation method for buried radioactive contamination based on an approximate three-dimensional (3D) linear attenuation model. Both simulation and experimental results have shown that the method has significantly improved depth profiling ability in both concrete and soil compared to existing remote techniques thereby increasing its range of application. The next section presents the derivation of the 3D linear attenuation model and the simulation and experimental setups. The results and discussions are presented in Section 3 and Section 4, respectively, while conclusions and future directions are presented in Section 5.

## 2. Materials and Methods

### 2.1. The Approximate 3D Linear Attenuation Model

**Figure 1:**
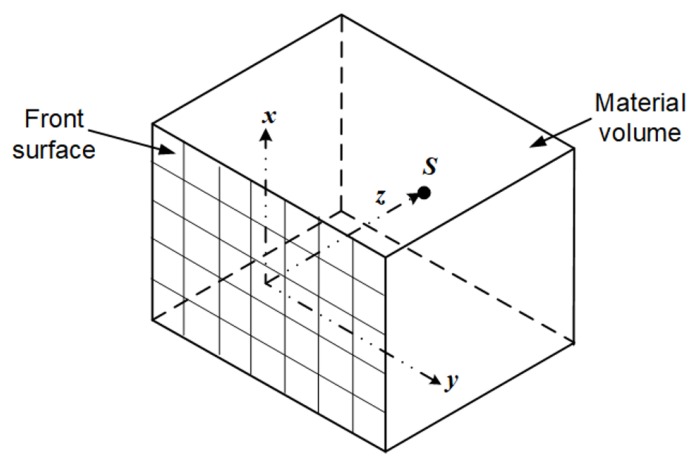
A point radioactive source buried in a section of a material.

Consider a point source *S* buried in a section of a material at a depth *z* from the front surface as shown in Figure 1. The intensity $I_{\left(x,y,z\right)}$ of the source measured by a collimated detector at any position on the *x*–*y* plane (i.e., the material surface) is given by:

(1) $$I_{\left(x,y,z\right)}=I_{\left(x,y,0\right)}e^{-\mu {\left(x^{2}+y^{2}+z^{2}\right)}^{\frac{1}{2}}},$$

where $I_{\left(x,y,0\right)}$ is the intensity at any position on the *x*–*y* plane when the source is at z=0 and μ is the linear attenuation coefficient. Equation (1) is the well known linear attenuation model [19] in 3D coordinates. Furthermore, Equation (1) can also be re-written as:

(2) $$I_{\left(x,y,z\right)}=I_{\left(x,y,0\right)}e^{-\mu z{\left(1+\frac{x^{2}+y^{2}}{z^{2}}\right)}^{\frac{1}{2}}}.$$

Expanding the index of the exponential term in Equation (2) using the binomial theorem and retaining only the first two terms of the binomial expansion results in:

(3) $$I_{\left(x,y,z\right)}\approx I_{\left(x,y,0\right)}e^{-\mu (z+\frac{x^{2}}{2z}+\frac{y^{2}}{2z})}.$$

However, it can be observed in Figure 1 that the intensity at the centre position of the *x*–*y* plane (i.e., x=y=0) is given by:

(4) $$I_{\left(0,0,z\right)}=I_{\left(0,0,0\right)}e^{-\mu z}.$$

Therefore, dividing Equation (3) by Equation (4) results in Equation (5), which can be rewritten as Equation (6):

(5) $$\frac{I_{\left(x,y,z\right)}}{I_{\left(0,0,z\right)}}\approx \frac{I_{\left(x,y,0\right)}}{I_{\left(0,0,0\right)}}e^{-\frac{\mu }{2z}\left(x^{2}+y^{2}\right)},$$

(6) $$\begin{array}{c} log_{e}\left(J_{\left(x,y,z\right)}\right)\approx -\frac{\mu }{2z}\left(x^{2}+y^{2}\right)+log_{e}\left(K_{\left(x,y,0\right)}\right),\\ \mathrm{where}:\;J_{\left(x,y,z\right)}=\frac{I_{\left(x,y,z\right)}}{I_{\left(0,0,z\right)}}\;\mathrm{and}\;K_{\left(x,y,0\right)}=\frac{I_{\left(x,y,0\right)}}{I_{\left(0,0,0\right)}}. \end{array}$$

Equation (6) is the approximate linear attenuation model of the intensity measured at any position on the *x*–*y* plane normalised by the intensity measured at the central position on the same plane. This is valid for $x^{2}+y^{2}<z^{2}$, which is the validity condition of the binomial expansion. Therefore, it can be deduced that, for a source buried at some depth *z*, the graph of $log_{e}\left(J_{\left(x,y,z\right)}\right)$ against $x^{2}+y^{2}$ for all $x^{2}+y^{2}<z^{2}$ should be a straight line passing through the origin since $log_{e}\left(K_{\left(x,y,0\right)}\right)=0$ at x=y=0. However, since *z* is not known, the normalised intensities from all the measured positions can be plotted and a weighted curve fitting method used to fit a straight line through the best points. The approximate depth of the radioactive source from the surface of the material can then be calculated from the slope of the fitted line. However, it is important to account for the dependence of the linear attenuation coefficient on the energy of the emitted photons. This can be done by using only gamma photons from a section of the measured energy spectrum over which the linear attenuation coefficient can be assumed to be constant. Theoretically, any region of the spectrum can be used since Equation (6) is a ratio of two spectra. However, the ideal region of interest is obviously the characteristic photopeak region of the buried radionuclide.

### 2.2. Monte Carlo Modelling and Simulation

**Figure 2:**
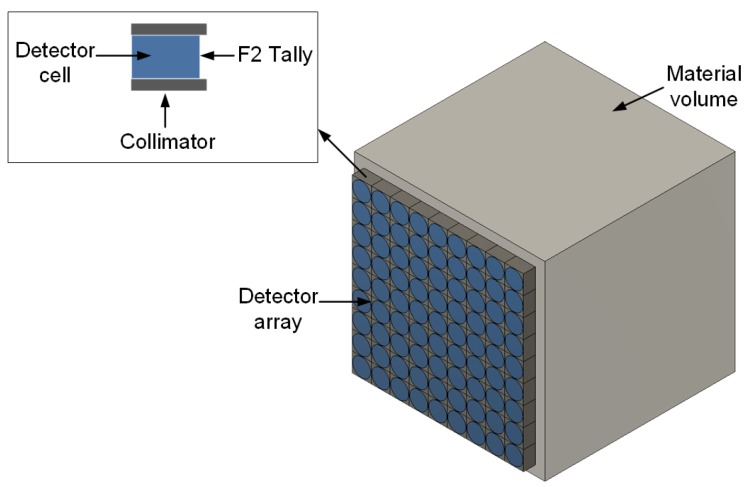
Sketch of the MCNPX simulation model.

In order to validate the derived model, Monte Carlo modelling and simulations were performed using MCNPX version 2.7 (Los Alamos National Laboratory, Los Alamos, NM, USA). MCNPX is a radiation transport code used to simulate the transportation and interaction of atomic particles in different media using Monte Carlo statistics [20]. A sketch of the MCNPX model used for the simulations is shown in Figure 2. It consists of an array of n×n detectors placed on the surface of a section of a material of uniform density where *n* depends on the grid (or detector) size and the total surface area to be measured. This configuration is equivalent to moving a single detector in n×n discrete locations across the material surface. Each detector (Figure 2 inset) is made up of a cylindrical detector cell surrounded by a 0.1 cm-thick square collimator. The MCNPX F2 tally was used to record all gamma photons crossing the front surface of the detector cell. The MCNPX F2 tally measures the average flux over a given surface area i.e., number of particles per cm${}^{2}$ [20]. In order to prevent crosstalk among neighbouring detectors, the collimator was set to be completely impenetrable by gamma photons.

Two radionuclides, namely: caesium-137 (Cs-137) and Co-60, were used in the simulations. These are by-products of the nuclear fuel cycle commonly encountered during decommissioning [18]. Both radionuclides were modelled as radioactive point sources. Furthermore, three different materials, namely: sand, ordinary dry concrete and high density concrete, were investigated. The properties of these materials are as shown in Table 1. Finally, for each material, a radionuclide was buried at varying depths ranging from 2 to 40 cm at 2 cm increments. At each depth, a total of 1E8 gamma particles were generated and the total number of gamma rays crossing each detector surface were recorded together with their corresponding energies.

### 2.3. Experiment Setup

**Figure 3:**
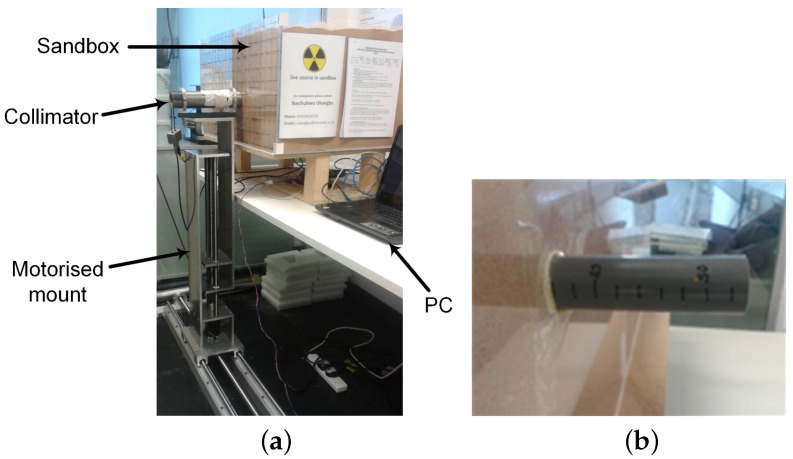
(**a**) Experiment setup; (**b**) graduated pipe for adjusting the distance of the source from the front of the sandbox.

The experiment setup is as shown in Figure 3a. It consists of a sandbox filled with fine silica sand in which a radioactive source was buried. The sandbox was constructed using 0.8 cm thick Perspex sheets (Direct Plastics Ltd., Sheffield, UK) because of its relative transparency to gamma radiation. This ensures that the scattering of the gamma radiation is almost exclusively due to the sand matrix. The radioactive source used was a sealed 392 kBq Cs-137 radioactive point source. The source was attached to one end of a graduated Ploy Vinyl Chloride pipe whose other end protrudes behind the sandbox (Figure 3b). This enables the distance of the source from the front of the sandbox (i.e., scanning surface) to be easily varied and the value read off from the pipe.

The detector used in the experiment consists of an organic liquid scintillator and a photomultiplier tube enclosed in a cylindrical aluminium case whose diameter is 3.5 cm and height is 9.13 cm. The organic liquid scintillator is the EJ-301 from Eljen Technology (Sweetwater, TX, USA) with a scintillation efficiency of 12,000 photons/MeV [22]. The entire detector assembly was placed inside the tungsten collimator shown in Figure 3a. The collimator is a hollow cylinder open at both ends with an internal diameter of 4 cm, thickness of 1 cm and length of 25 cm. The use of the tungsten collimator was to ensure that only gamma photons within the detector’s field of view are detected. However, since no material can provide 100% shielding, some gamma photons are still able to penetrate through the walls of the collimator. For instance, the collimator has a penetration of 14.4% at 662 keV for photons striking the curved surface at $90^{{}^{\circ}}$. However, it is obvious from the experiment setup that none of the photons leaving the sandbox will strike the curved surface of the collimator at $90^{{}^{\circ}}$. Therefore, assuming a maximum striking angle of $45^{{}^{\circ}}$, a photon will travel a minimum thickness of 1.4 cm resulting in a significantly lower penetration of 6.4%. Finally, the collimator was mounted on a custom fabricated motorised mount to enable automated and accurate positioning at specified *x*–*y* coordinates.

During the experiment, the source was positioned at the centre of the scanning surface while its distance from this surface was varied from 2 cm to 14 cm at 2 cm intervals. At each distance, the spectrum of the source was measured on a total scan area of 28×28 cm${}^{2}$, which was divided into 4×4 cm${}^{2}$ cells where a cell represents the area covered by the detector at that position. This yields a total of 49 spectra per distance. Finally, a scanning time of 10 min per position was used in the experiment.

## 3. Results

### 3.1. Simulation Results for Cs-137 Buried in Sand

**Figure 4:**
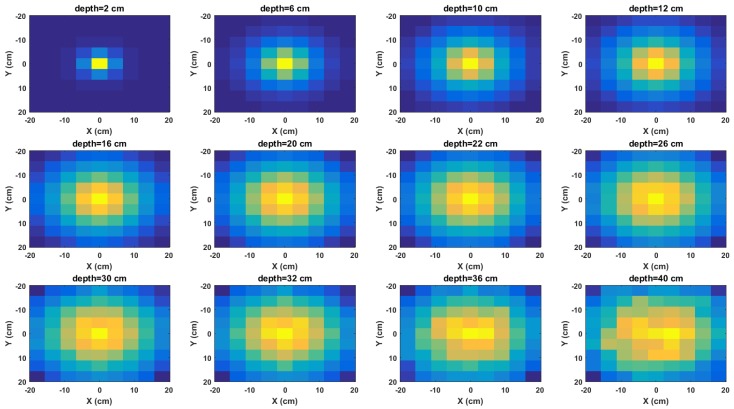
Normalised radiation images of Cs-137 buried in sand for selected depths. From left to right: 2 cm, 6 cm, 10 cm, 12 cm, 16 cm, 20 cm, 22 cm, 26 cm, 30 cm, 32 cm, 36 cm and 40 cm.

The normalised radiation image of the Cs-137 point source buried in sand and acquired using a cell size of 4×4 cm${}^{2}$ and a maximum scan area of 36×36 cm${}^{2}$ are shown in Figure 4. The intensity of each pixel is the number of gamma photons with energy between 640 and 662 keV detected by the detector at that position. This part of the energy spectrum was chosen because it contains the characteristic photopeak of Cs-137. As expected, the intensities of the images gradually spreads out to neighbouring pixels as the depth of the source increases. This is mainly due to increasing spreading of the emitted gamma rays and scattering of the photons by the sand matrix. This makes the photons be detected by an increasing number of detectors as the source depth increases.

**Figure 5:**
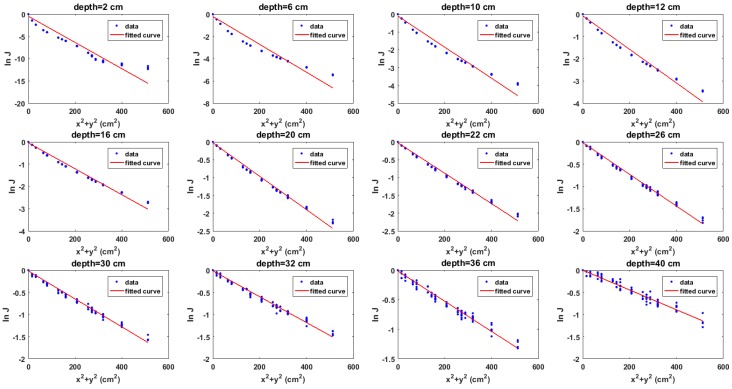
Plots of model for Cs-137 buried in sand for selected depths. From left to right: 2 cm, 6 cm, 10 cm, 12 cm, 16 cm, 20 cm, 22 cm, 26 cm, 30 cm, 32 cm, 36 cm and 40 cm.

The graph of the model (i.e., Equation (6)) for each of the images in Figure 4 are shown in Figure 5. As predicted by the model, it can be observed that the data points approach a straight line with negative slope as the depth increases. This is because more cells meet the validity condition of the binomial expansion at lower depths. Furthermore, the effects of attenuation at lower depths can be observed where the points become increasingly scattered at random about the straight line.

In order to estimate the approximate depth from the slope of the fitted line in the model plots (Figure 5), the mean linear attenuation coefficient for sand at 640–662 keV is required. This was calculated using (7), where ρ is the density of sand, $\mu_{m,i}$ is the mean mass attenuation coefficient between 640–662 keV for each element *i* of the sand mixture and $W_{i}$ is the weight ratio of each element *i* of the sand mixture. The elements that constitute the sand mixture, their weight ratios and mass attenuation coefficients were obtained from standard published tables [23]:

(7) $$\mu =\rho \sum_{i=1}^{n}\mu_{m,i}W_{i}.$$

**Figure 6:**
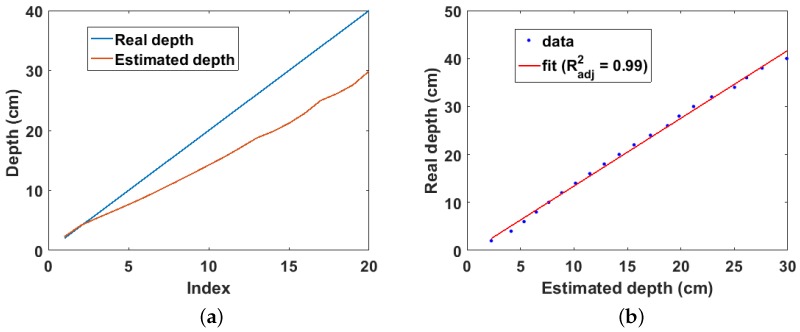
(**a**) Real and estimated depths for Cs-137 buried in sand. Index is the position of each depth value in the depth array; (**b**) linear fit of real and estimated depth for Cs-137 buried in sand.

The estimated and real depths for the Cs-137 point source buried in sand are shown in Figure 6a. It can be observed that the real depth is well approximated by the estimated depth for depths of up to 5 cm. However, the estimated depth increasingly deviates from the real depth at lower depths. This seems counter intuitive at first glance because it is expected that more cells should fulfil the validity condition of the binomial expansion at lower depths; consequently, lower depths should be better approximated than shallow depths. However, this increasing error at lower depths is as a result of the exponential increase in the truncation error caused by selecting only the first two terms of the binomial expansion. However, of more practical importance is the linear relationship between the real and estimated depths as shown in Figure 6b. This shows that the real depth can be predicted from the estimated depth by a simple calibration.

#### 3.1.1. Effects of Scan Area and Grid Cell Size

**Figure 7:**
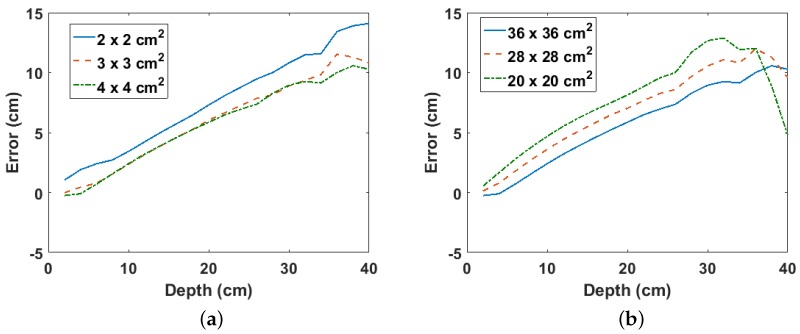
(**a**) Error per depth for different cell sizes for Cs-137 buried in sand; (**b**) error per depth for different scan areas for Cs-137 buried in sand.

The two parameters that affect the estimated depth using the proposed method are the size of the grid cells and the total scan area. This is because they determine the depth beyond which the binomial expansion used in the derivation of (6) becomes valid. For instance, smaller cell sizes increase the number of cells that meet this validity condition, thereby yielding more valid points, which increases the accuracy of the fitted line from which the approximated depth is estimated. However, as can be observed from Figure 7a, larger cell sizes result in smaller errors in the estimated depth compared to smaller cell sizes. This suggests that the number of gamma photons detected per cell is an important factor because larger cell sizes (i.e., larger detectors) detect more gamma rays per cell compared to smaller cell sizes. However, cell sizes beyond 3×3 cm${}^{2}$ yield only marginally smaller errors. Figure 7b shows the error per depth for different scan areas using a fixed cell size of 4×4 cm${}^{2}$. As expected, larger scan areas yield smaller errors with a consistent linear relationship with the depth. However, a sudden drop in the error for depths above 30 cm can be observed for the smaller scan area of 20×20 cm${}^{2}$. This is probably due to error in the Monte Carlo statistics as the same trend is not observed for Co-60 buried in sand (see Section 3.3). Finally, though a larger scan area will yield better estimates, practical limitations such as available space and time may place a limit on the maximum surface area that can be scanned.

### 3.2. Simulation Results for Cs-137 Buried in Concrete

**Figure 8:**
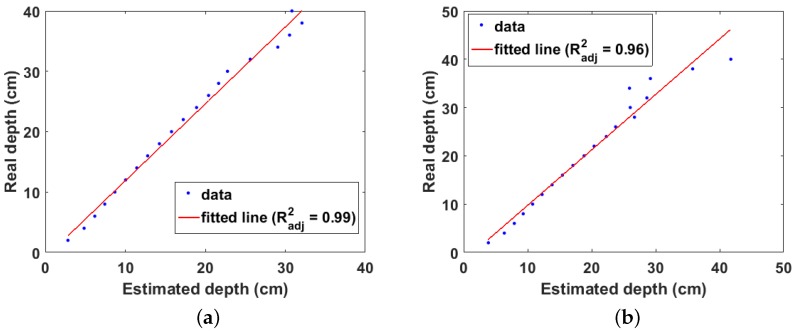
(**a**) Linear fit of estimated and real depth for Cs-137 buried in concrete of density = 2.18 g cm${}^{-3}$; (**b**) linear fit of estimated and real depth for Cs-137 buried in concrete of density = 3.35 g cm${}^{-3}$.

Figure 8a,b shows the linear fit of the estimated and real depths for the two types of concrete, respectively. The effects of attenuation of the emitted gamma rays in both types of concretes can be observed. This corresponds to the region where the data points begin to lose their linearity. Furthermore, as expected, this loss of linearity is more pronounced in the higher density concrete. This shows that Equation (6) correctly models the attenuation behaviour of gamma rays in different materials.

### 3.3. Simulation Results for Co-60 Buried in Sand and Concrete

**Figure 9:**
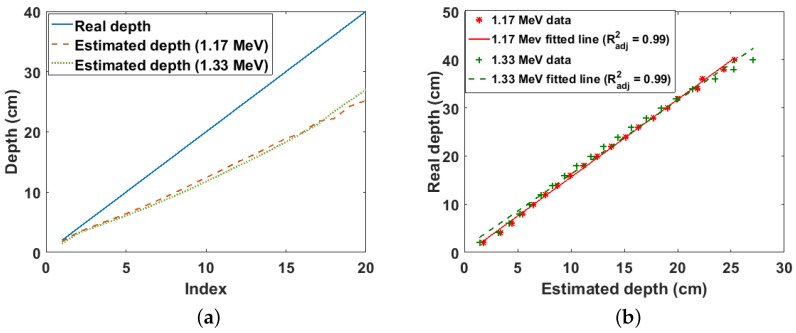
(**a**) Eeal and estimated depths for Co-60 buried in sand. Index is the position of each depth value in the depth array; (**b**) linear fit of real and estimated depth for Co-60 buried in sand.

**Figure 10:**
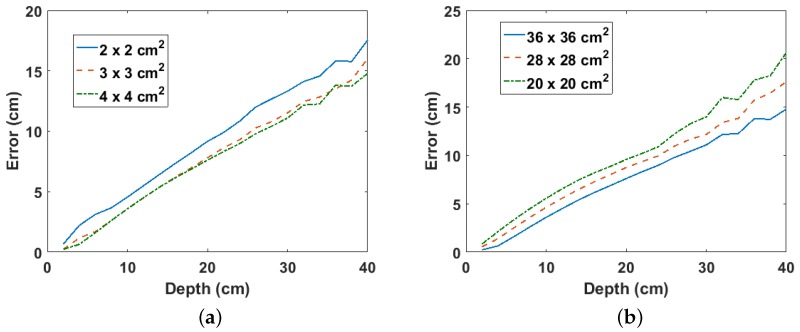
(**a**) Error per depth for different cell sizes for Co-60 buried in sand; (**b**) error per depth for different scan areas for Co-60 buried in sand.

Co-60 is known to have two prominent photopeaks at 1.17 MeV and 1.33 MeV on its energy spectrum. The results using photon counts from both photopeak regions are shown in Figure 9a,b. The same error pattern in the estimated depth as seen in Cs-137 can also be observed. This proves the consistent behaviour of the proposed model. Furthermore, there is no significant difference in the estimated depth using photon counts from either photopeaks. This is because the probability of Co-60 emitting gammas with either energies is almost equal in addition to the fact the difference between both energies is not substantial. It can be observed in Figure 10a,b that larger cell sizes and scan areas yield better estimates similar to the results obtained for Cs-137. This shows that the behaviour of these parameters is independent of the energy of the gamma rays.

**Figure 11:**
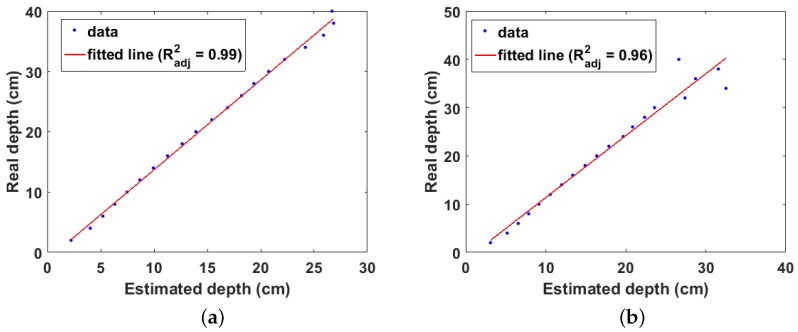
(**a**) linear fit of estimated and real depth for Co-60 buried in concrete of density = 2.18 g cm${}^{-3}$; (**b**) linear fit of estimated and real depth for Co-60 buried in concrete of density = 3.35 g cm${}^{-3}$.

The linear fit of the estimated and real depths for Co-60 buried in the two types of concrete are shown in Figure 11. As can be observed, the depth at which there is significant uncertainty in the estimated depth due to attenuation is lower compared to the case of Cs-137 (Figure 8). This is as expected because attenuation decreases with increasing gamma energy. Consequently, Co-60 should have a higher maximum detectable depth compared to Cs-137 when buried in the same material.

### 3.4. Experiment Results

**Figure 12:**
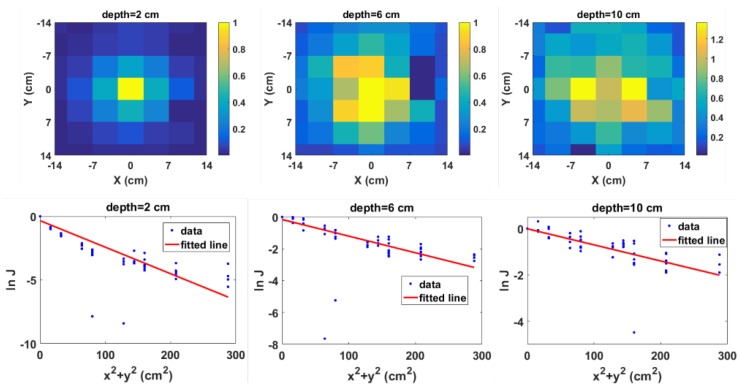
Normalised 2D radiation image using photons from the Compton peak region (**top row**) and corresponding model plot (**bottom row**) for selected depths from the experiment.

As pointed out in Section 2.1, any region of the measured energy spectrum can theoretically be used in the depth estimation. This is especially useful for detectors that cannot detect the characteristic photopeak of the entrained radionuclide such as the detector used in the experiment. Therefore, gamma photons from the Compton peak were used in estimating the depth from the measured spectra. This corresponds to the energy range between 451 to 500 keV. The radiation images and corresponding model plots for selected source depths from the experiment are shown in Figure 12. The same trend seen in the simulation results can be observed. However, one or two outlier data points due to measurement errors can be seen in the model plots (Figure 12 bottom row).

**Figure 13:**
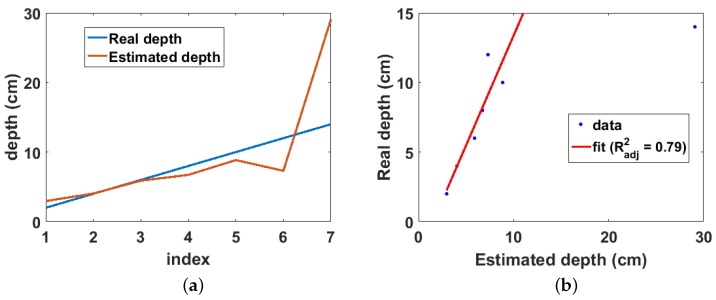
(**a**) real and estimated depths from experiment. Index is the position of each depth value in the depth array; (**b**) linear fit of real and estimated depths from experiments.

The real and estimated depths from the experiment are shown in Figure 13a. It can be observed that the real depth is well approximated by the estimated depth up to 10 cm. Beyond 12 cm, the effect of attenuation becomes significant, resulting in large errors in the estimated depth. This is also observed in the linear fit between the real and estimated depths (Figure 13b), where a depth of 29 cm was estimated from the model when the real source depth is 14 cm. Due to this large error, this data point was not included in fitting the data. Therefore, the maximum detectable depth for the experiment setup is 12 cm with an adjusted R-squared value of 0.79. However, this depth is lower compared to that obtained from the simulation results. This can be attributed to three main factors, the first of which is the weak activity of the sealed Cs-137 point source used in the experiment. Secondly, unlike the simulation, the experiment used a realistic collimator therefore, its estimates will be affected by the uncertainties caused by photons that penetrate through the walls of the collimator; and, finally, the simulation used photons from the photopeak region which are higher energy and number compared to photons in the Compton peak region and are therefore less susceptible to attenuation.

## 4. Discussion

The simulation and experiment results show that the proposed technique has significant advantages compared to existing remote contamination depth estimation methods. First, it has significantly higher maximum detectable depth, thereby increasing its range of applications. For instance, in a recent technical report [24], it was identified that significant internal contamination in pipes buried up to 50 cm deep can be detected on the ground surface using a radiation detector. The proposed method will enable non-intrusive monitoring and characterisation of such pipelines through remote 3D localisation of internal contamination. Secondly, the experiment results showed that the method can be used with non-spectroscopic gamma radiation detectors such as plastic scintillators [25]. This is advantageous because these type of detectors are cheaper compared to other types detectors.

The main limitation of the proposed method is in the estimation of the linear attenuation coefficient of the material in which the radionuclide is buried. This is because it requires foreknowledge of the mass attenuation coefficient and density of the entraining material (see Equation (7)). However, Table 2 shows that the average mass attenuation coefficient for a given energy range is relatively constant for different materials. Therefore, the problem of estimating the linear attenuation coefficient is reduced to that of finding only the density of the entraining material. While a table of the densities of common materials can be prepared, such a solution does not take into account the changes the material may have undergone overtime due to environmental factors. Therefore, a better solution will be to integrate data from other non-intrusive techniques such as ground penetrating radar as proposed in [26]. This multisensor data fusion solution will enable real-time determination of the entraining material properties and also potentially improve the accuracy of the estimated depth.

## 5. Conclusions

A novel method for remote depth estimation of radioactive contamination has been presented. The method is based on a derived approximate 3D linear attenuation model and exploits the information obtained from multiple measurements of the intensity of the radiation on the surface of the material in which the contamination is buried. Results from simulations and experiments of Cs-137 and Co-60 contaminations in sand and concrete showed significantly improved remote depth estimation capabilities compared to existing methods. Finally, the proposed method will significantly enhance the non-intrusive characterisation of buried radioactive wastes commonly encountered during the decommissioning of nuclear sites and facilities. 

## Figures and Tables

**Table 1:** Densities and elemental composition of the three materials used in the simulation. The information was obtained from [21].

Elements	Weight Fraction		
	Sand	Ordinary Concrete	High Density Concrete
	(*density* =1.7 g cm${}^{-3}$)	(*density* =2.18 g cm${}^{-3}$)	(*density =* 3.35 g cm${}^{-3}$)
H	0.007833	0.004000	0.003585
C	0.003360	-	-
O	0.536153	0.482102	0.311622
Na	0.017063	0.002168	-
Mg	-	0.014094	0.001195
Al	0.034401	0.069387	0.004183
Si	0.365067	0.277549	0.010457
K	0.011622	0.013010	-
Ca	0.011212	0.080229	0.050194
Fe	0.013289	0.057461	0.047505
S	-	-	0.107858
Ba	-	-	0.463400
	1.000000	1.000000	1.000000

**Table 2:** Average mass attenuation coefficients for different materials at the photopeak region of Cs-137 and Co-60 calculated from [23].

Material	Cs-137	Co-60	
	600–700 keV	1.1–1.2 keV	1.3–1.4 keV
Sand	0.0800	0.0606	0.0557
Concrete 1 (2.18 g cm${}^{-3}$)	0.0795	0.0602	0.0553
Concrete 2 (3.35 g cm${}^{-3}$)	0.0809	0.0576	0.0526
